# Low Genetic Diversity and Strong Geographical Structure of the Critically Endangered White-Headed Langur (*Trachypithecus leucocephalus*) Inferred from Mitochondrial DNA Control Region Sequences

**DOI:** 10.1371/journal.pone.0129782

**Published:** 2015-06-09

**Authors:** Weiran Wang, Yu Qiao, Wenshi Pan, Meng Yao

**Affiliations:** 1 School of Life Sciences, Peking University, Beijing, China; 2 Chongzuo Biodiversity Research Institute of Peking University, Guangxi, China; University of British Columbia Okanagan, CANADA

## Abstract

Many Asian colobine monkey species are suffering from habitat destruction and population size decline. There is a great need to understand their genetic diversity, population structure and demographic history for effective species conservation. The white-headed langur (*Trachypithecus leucocephalus*) is a Critically Endangered colobine species endemic to the limestone karst forests in southwestern China. We analyzed the mitochondrial DNA (mtDNA) control region sequences of 390 fecal samples from 40 social groups across the main distribution areas, which represented one-third of the total extant population. Only nine haplotypes and 10 polymorphic sites were identified, indicating remarkably low genetic diversity in the species. Using a subset of 77 samples from different individuals, we evaluated genetic variation, population structure, and population demographic history. We found very low values of haplotype diversity (*h* = 0.570 ± 0.056) and nucleotide diversity (*π* = 0.00323 ± 0.00044) in the hypervariable region I (HVRI) of the mtDNA control region. Distribution of haplotypes displayed marked geographical pattern, with one population (Chongzuo, CZ) showing a complete lack of genetic diversity (having only one haplotype), whereas the other population (Fusui, FS) having all nine haplotypes. We detected strong population genetic structure among habit patches (*Φ*_ST_ = 0.375, *P* < 0.001). In addition, the Mantel test showed a significant correlation between the pairwise genetic distances and geographical distances among social groups in FS (correlation coefficient = 0.267, *P* = 0.003), indicting isolation-by-distance pattern of genetic divergence in the mtDNA sequences. Analyses of demographic history suggested an overall stable historical population size and modest population expansion in the last 2,000 years. Our results indicate different genetic diversity and possibly distinct population history for different local populations, and suggest that CZ and FS should be considered as one evolutionarily significant unit (ESU) and two management units (MUs) pending further investigation using nuclear markers.

## Introduction

Genetic diversity plays a critical role in ecological adaptation and long-term survival of a species. Loss of genetic variation in wild populations is often associated with increased extinction risk due to reduced individual fitness and weakened resistance to natural and anthropogenic disturbances [1–3]. Genetic information can be valuable in designing conservation breeding [4], managing reintroductions [5], defining conservation units [6], determining conservation priorities [7], and evaluation of conservation effectiveness [8]. Therefore, genetic monitoring of wild populations has increasingly become an integrated component of conservation and management of endangered species [9,10].

Asian colobines are a morphologically and evolutionarily diverse group of primates including 55 species in seven genera, and mainly found in South and Southeast Asia [11,12]. Many of the species suffer from human-caused habitat destruction, fragmentation, and population decline and are threatened with extinction [12]. The white-headed langur, *Trachypithecus leucocephalus*, is a flagship species for conservation of the limestone karst forest ecosystem that belongs to the Indo-Burma biodiversity hotspot [13]. With a total of less than 1000 individuals remaining in the wild, it is classified as Critically Endangered on the IUCN Red List [14] and listed as a Class I national protected species of China. The species is endemic to the limestone forests in Guangxi Province, southwestern China, within a narrow range bounded by Zuojiang River, Mingjiang River, and Sifangling Mountains. Its suitable habitat, the limestone hills, is only about 360 km^2^ and in patchy distribution [15,16]. The current range includes five isolated areas varying from 20 to 100 km^2^ in size [15–17], ranging from near local extinction to over 500 individuals living in each area [17]. These areas are 10–80 km apart and separated by rivers, farmlands, human settlements, and roads. Land exploitation for agricultural purposes has further fragmented larger habitat areas into smaller patches or even isolated hills. Functional continuity among and within these areas with regard to the white-headed langur’s genetic connectivity has not been assessed, and the extents of individual migration and gene flow at various spatial scales remain unknown.

Little is known about the species’ historical distribution and population size before the 1980s. The total distribution range for the species shrank by almost 80% during the last two decades of the 20th century, along with extirpation of local populations in small habitat patches [15,16]. One study reported nearly 60% population size reduction in Fusui (FS) area, where the largest extant population resides, from 1987 to 1997 [18]. Other distribution areas were likely to have experienced population declines during the same period of time. Habitat destruction and fragmentation by human development and poaching were possibly the main reasons for severe population decline during this period of time [15,19,20].

Genetic data on the white-headed langur are very limited, and large-scale genetic studies have been lacking. One study found very low genetic diversity in the species in comparison with a closely related species, the François' langur (*Trachypithecus francoisi*), using mitochondrial DNA (mtDNA) sequences from 54 fecal samples [21]. No data on population structure or genetic differentiation are yet available in the species. Appropriate conservation strategies demand accurate evaluation of the present and past population genetic parameters. In this study, we collected fecal samples noninvasively from 37% of all known social groups across the two main distribution areas, FS and Chongzuo (CZ), and analyzed haplotypic variation at the mtDNA control region. Our main aims were: (1) to assess genetic diversity in the local and total populations, (2) to investigate population structure and genetic differentiation among and within the local populations, (3) to infer population demographic history, and (4) to make management recommendations for conservation of the species’ genetic variation.

## Materials and Methods

### Study area and species information

Most of the current distribution range of the white-headed langur is included in the Chongzuo National Nature Reserve (22°10′43″-22°36′55″ N, 107°16′53″-107°59′46″ E; total area 256 km^2^) in Guangxi Province, China. The landscape is karst topography characterized by limestone peak hills and valleys with evergreen and semi-evergreen forests. The study areas are dominated by agricultural activities. Even within the Nature Reserve, most flatlands and valleys have been exploited for agricultural purposes, and the mean human population density is 220 per km^2^. White-headed langurs inhabit the hills amid the farmlands, roads, and human settlements.

**Fig 1:**
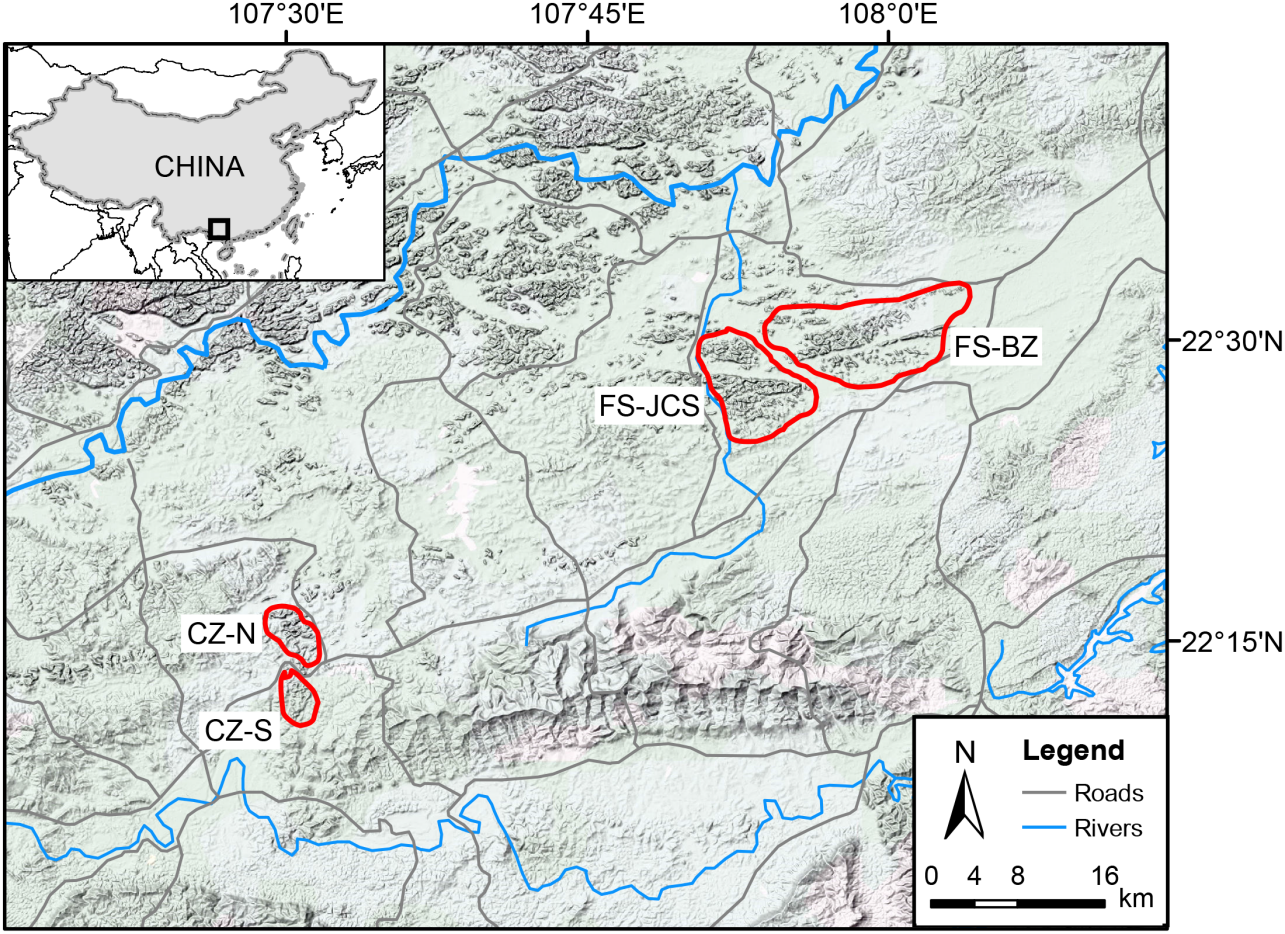
Map of study area in Chongzuo National Nature Reserve, Guangxi, China. Circled areas represent sampling range of the white-headed langur. FS- JCS, Fusui-Jiuchongshan; FS-BZ, Fusui-Buzun; CZ-N, Chongzuo-North; CZ-S, Chongzuo-South.

The most recent species-wide population census counted a total of 937 individuals in 120 social groups [17]. Over 90% of the extant population inhabits FS (area size: 95km^2^) and CZ (area size: 22 km^2^) areas within the Nature Reserve. A total of 552 individuals in 77 groups and 306 individuals in 33 groups were found in FS and CZ, respectively [17]. FS is divided into several habitat patches by farmlands, roads and rivers, the largest two being Jiuchongshan (FS-JCS) and Buzun (FS-BZ). CZ area is transected by a national highway and several limestone quarries into the north (CZ-N) and south (CZ-S) patches (Fig 1).

A reproductive social group of the species usually consists of on average 11.7 individuals (range 3–30), including one adult male, 5.1 adult females (range 1–14), and immatures [22]. All-male and one-male groups are also observed but less common. No other colobine species inhabits the study area.

### Sample collection

Social groups of wild white-headed langurs use rock ledges and caves on limestone cliffs as sleeping sites at night [23,24], and their feces accumulate on the ground underneath such sites. Fecal sample collection was conducted at the ground level during the daytime when the langurs were away from the sleeping sites.

We collected fecal samples in FS and CZ in January-April from 2012 to 2014. Our sampling locations covered most of the species’ current range in FS and CZ. We collected from almost the maximum number of groups that could be sampled in the study areas, since unsampled groups mostly used sleeping sites at inaccessible locations, such as cliffs over 100 meters above the ground level. Fresh fecal samples were selected based on the color, smoothness and moisture level of the surface. A lighter colored, smooth and moist fecal pellet was generally less than three days old and preferable for DNA isolation. Fecal samples were stored in 95–100% ethanol at ambient temperature in the field for up to three weeks, and transferred to -20 or -80°C upon arrival in the laboratory.

### Extraction of DNA

Extraction of DNA from fecal samples was conducted using a 2CTAB/PCI method [25], which had higher DNA extraction yields and subsequent amplification success from feces of vegetarian primate species in comparison with the QIAamp DNA Stool Mini Kit (Qiagen) [25]. Approximately 200 mg of feces taken from the surface of a sample was used in each extraction. We added an absorption step with potato starch (0.25 g) following the initial lysis step to absorb PCR inhibitors in the extracts [26,27]. The final DNA was dissolved in 200 μl TE buffer and stored at -20°C. An extraction negative control containing no feces was included in each extraction run to check for contamination. We further purified some DNA extracts using a DNA purification kit (EasyPure PCR Purification Kit, TransGen Biotech, China) to improve amplification. Concentrations of DNA were determined using a NanoDrop 2000 spectrophotometer (Thermo Scientific) and by agarose gel electrophoresis.

### Molecular sex identification

Molecular sex identification was done by PCR using a single pair of primers for sex chromosome-linked DEAD-box gene, which produces one fragment (the X fragment) from female DNA, and two fragments (the X and Y fragments) with about 30 bp difference in length from male DNA in human and many nonhuman primate species [28]. PCR were carried out in a total volume of 10 μL, including 10–20 ng DNA, 0.4 μg/μL BSA, 0.8 mM dNTPs, 0.5μM of each primer, 0.5 U Taq polymerase (rTaq, Takara Biotechnology, Japan), 1 × buffer (containing 1.5 mM MgCl_2_), and ddH_2_O. The conditions for PCR were predenaturing at 94°C for 3 min; followed by 35 cycles of denaturing at 94°C for 30 s, annealing at 62°C for 40 s, and extension at 72°C for 1 min; and a final extension at 72°C for 10 min at the end. The PCR products were separated and visualized on 8% polyacrylamide gels. The reliability of this molecular method for sex identification was first verified using fecal samples of known sex that were collected following witnessed defecation of 21 individuals (5 males and 16 females), and by sequencing the PCR products. The amplification patterns were consistent with the prediction based on the sex of the samples, and the sequencing results confirmed that the amplified fragments were of the DEAD-box gene from a nonhuman primate. The X and Y fragment sequences mapped onto human DEAD-box gene on chromosome X and Y (GenBank Accession No. NG_012830.1 and NG_012831.1) with 98% and 96% identity, respectively. Since the white-headed langur was the only primate species that we extracted DNA from in our laboratory, we were confident that the amplified sequences were from this species.

For sex identification of fecal samples, we followed a procedure modified from the protocol proposed by Eriksson et al. [29] for determining homozygous (female) and heterozygous (male) nuclear loci. With maximum five repetitions of successful amplications (i.e. at least one of the X or Y fragment was amplified), samples from which the Y fragment was amplified in two or more independent PCR were determined to be males, and samples from which only the X fragment was amplified in at least four PCR were determined to be females. If a sample failed sex identification, DNA was re-extracted and amplified until an unambiguous sex was determined. Samples that also failed sexing by re-extraction were designated as sex ‘unknown’.

Other molecular sexing systems including the AMELX-AMELY [30], amelogenin X-SRY [31], UTX-UTY [32] and several other systems that we designed were also tested, but either failed to amplify in PCR or generated products of similar sizes in male and female samples.

### Mitochondrial DNA (mtDNA) sequencing

A total of 1213 bp of mtDNA sequences were amplified in PCR with primer pair P3 and P87 (S1 Table), which include the entire control region and the *tRNA* ^*Phe*^ gene, and partial sequences of *tRNA* ^*Pro*^ and *12S rRNA* genes. The PCR reagents and conditions were similar as in DEAD-box gene amplification, except with different primers, annealing at 55°C for 30 s, and in a total volume of 50μL. The PCR products were checked on 1.5% agarose gels. If PCR failed to amplify the full length product, we used three primer pairs (P3-P4, P78-P79, and P82-P87; S1 Table) to amplify overlapping fragments of 400–600 bp in length that collectively encompassed the full length sequence. We obtained the full length sequences for 271 (67%) samples. A 574 bp fragment (primer pair P3-P470; S1 Table) covering the hypervariable region I (HVRI) of the control region was amplified for all samples (see Results).

Successful amplifications were purified using a gel extraction kit (Agarose Gel Extraction Kit, Biomed, China), and sequenced on an ABI PRISM 3730 Genetic Analyzer with the BigDye Cycle Sequencing Kit (Applied Biosystems) for both strands. Sequencing results were checked by eye, and shorter fragments were manually assembled into full sequences in SeqMan II 5.01 (DNAStar Inc., Madison, WI). Negative controls containing no DNA templates were included in PCR to check for contamination.

We also amplified and sequenced 10% randomly selected DNA samples from independent extractions to verify sequencing results and exclude contamination. All PCR amplified a single band of predicted size, and no highly divergent sequences were detected. The amplified sequences matched the mtDNA control region sequences of the François' langur (GenBank Accession No. KJ174502.1) with 94% identity. To further exclude the possibility of contamination by nuclear insertions of mtDNA fragments (numts; [33]), we conducted the dilution experiments [34] with 10% of all samples. This method relies on the fact that there are more copies of mtDNA than nuclear DNA in a cell, and potential numts can be detected by diluting DNA extracts to concentrations at which only the mtDNA can be sufficiently amplified. We co-amplified a mtDNA fragment (P3-P4; S1 Table) and a microsatellite locus (fragment length around 300 bp) in PCR. PCR products of the mtDNA fragment were sequenced and compared with the sequences from undiluted DNA samples. All sequences derived from the same sample were identical. Therefore we concluded that there were no numts in our sequencing results.

By direct sequencing of PCR products, we found that all samples amplified a single sequence except samples of one social group from FS-BZ, which yielded two sequences differing at one site. This was most likely to represent a case of mitochondrial heteroplasmy, where more than one mitochondrial genome is present in an organism and can co-transmit [35]. Since we found no samples from any groups with only one of the two haplotypes, these two sequences appeared to be co-transmitted. Because software for population genetic analyses generally does not accommodate the situation of heteroplasmy or allow degenerate sequences, we treated these two sequences as two distinct haplotypes each occurring in half the number of heteroplasmic samples. As this treatment only concerned two individuals in the reduced sample set of 77 used in genetic data analyses (see below), it had very little effect on the results in comparison to treating the two sequences as a single haplotype.

### Data analyses

#### Genetic diversity of the mtDNA control region

The mtDNA sequences were aligned and edited using the software CLUSTAL X version 2.1 [36]. We trimmed the sequences to 350 bp for data analyses (see Results). To avoid repeated sampling of the same individual, we used one sequence of each haplotype and sex from each social group in the following analyses. This sampling strategy resulted in 77 sequences, representing the maximum number of sequences from different individuals based on sex and mtDNA haplotype in the total samples. Genetic diversity parameters including the number of polymorphic sites (*s*) and haplotypes (*H*), haplotype diversity (*h*), and nucleotide diversity (*π*) [37] of the HVRI sequences were estimated in DNASP version 5.10.01 [38]. The most appropriate model of nucleotide substitution was the HKY model as suggested by the Corrected Akaike Information Criterion (AICc) [39] and hierarchical likelihood-ratio tests (hLRTs) in the software ModelGenerator [40]. This model or its best approximation available was used in the analyses where one was applied.

#### Population genetic structure

A median-joining network of the HVRI haplotypes was constructed using the software NETWORK version 4.6.1.2 [41] to show mutational relationships and the geographical distribution of the haplotypes.

Population genetic structure and differentiation among populations was evaluated by treating the geographic area of sample collection as an a priori defined local population (FS-JCS, FS-BZ, CZ-N, and CZ-S). Because CZ-N and CZ-S samples showed only a single haplotype, we combined their data and considered CZ as a single population in the analyses. Geographical differentiation of the HVRI haplotypes was computed using Analysis of Molecular Variance (AMOVA) as implemented in the software ARLEQUIN version 3.5.1.2 [42], and the significance of the observed AMOVA was assessed using the null distribution generated from 10,000 random permutations. Pairwise population fixation indices *Φ*_ST_ between the populations were also estimated in ARLEQUIN with 10,000 random permutations. The significance threshold of multiple pairwise *Φ*_ST_ analyses was adjusted using sequential Bonferroni correction [43].

Genetic differentiation due to isolation by distance among social groups of FS (including both FS-JCS and FS-BZ) was assessed by the Mantel test [44], which analyzes the correlation between genetic distances measured as *Φ*_ST_/(1- *Φ*_ST_) [45] and the geographical distances using ARLEQUIN. Pairwise *Φ*_ST_ among social groups of the FS population were estimated in ARLEQUIN, and the geographical distances between the sampling sites were calculated online at http://www.gpsvisualizer.com/calculators#distance. The CZ population was excluded from this analysis due to its lack of within-population genetic diversity and a relatively large geographical distance from FS. Statistical significance was calculated by 10,000 iterations at 95% confidence interval (CI).

#### Population demographic history

Historical changes in the population size of the white-headed langur were assessed by several methods. First, we conducted the neutrality tests including the Tajima’s *D* [46] and Fu’s *F*_S_ tests [47] in ARLEQUIN with 10,000 permutations to test for statistical significance. Significantly negative values generated by these tests of mtDNA sequences suggest population expansion. Second, we estimated growth rate (*g*) and mutation-scaled effective population size (*θ*) using the maximum-likelihood method in the software package LAMARC 2.1.10 [48] with the F84 model (the best approximation of the HKY model in the software). We applied 10 initial Markov chain Monte Carlo (MCMC) of 4,000 steps each and five final MCMC of 400,000 steps, each sampled every 20 steps. Five independent runs were conducted to ensure stability and convergence of parameter estimation. The starting value was set as Watterson’s *θ*_W_ [49] for estimation of *θ*, and zero for *g*. The first 1,000 genealogies were discarded as burn-in. Female effective population size (*N*_*ef*_) can be estimated by *N*_*ef*_ = *θ*/2*μ*, where *μ* is the mutation rate per site per generation and can be calculated using a mutation rate of 0.164 per nucleotide per million years estimated for the HVRI sequences in apes and human [50]. Third, we inferred population expansion patterns using the mismatch analysis [51] in DNASP with 10,000 bootstrap replicates. A recent population growth is expected to show a unimodal and smooth distribution of pairwise differences between sequences. Finally, to estimate the population size changes through time, we conducted Bayesian Skyline Plot (BSP) analyses [52] implemented in the software BEAST version 1.8.1 [53]. The analyses were run using a piecewise constant Bayesian Skyline prior, a strict clock model and a standard substitution rate of 0.164 per nucleotide per million years [50]. We ran MCMC of 6,000,000 steps, sampling every 1,000 steps. The first 10% were discarded as burn-in. The analysis was repeated three times with different seed numbers to check for convergence and mixing in LogCombiner version 1.8.1, and the effective sample size (ESS) for each parameter typically exceeded 200. Skyline plots were visualized using TRACER version 1.6.

We estimated the generation time for female white-headed langurs to be around 10 years, based on the average female age at first birth being 5–6 years [54] and approximately 20 years at the last birth in the species (W. Pan, personal observations).

### Ethics Statement

The research complied with protocols approved by the Chongzuo National Nature Reserve for the White-Headed Langur of Guangxi Province, and adhered to the legal requirements of China.

## Results

### Sample collection

We collected a total of 402 fecal samples from 41 social groups, including 216 samples from 20 groups of FS-JCS, 29 samples from 3 groups of FS-BZ, 132 samples from 14 groups of CZ-N, and 25 samples from 4 groups of CZ-S. Our sampling covered 37% of the known groups in FS and CZ, and one-third of all groups of the total extant population. The number of samples per group was 9.8 ± 5.8 (mean ± SD, range: 1–25).

### Molecular sex identification

**Fig 2:**
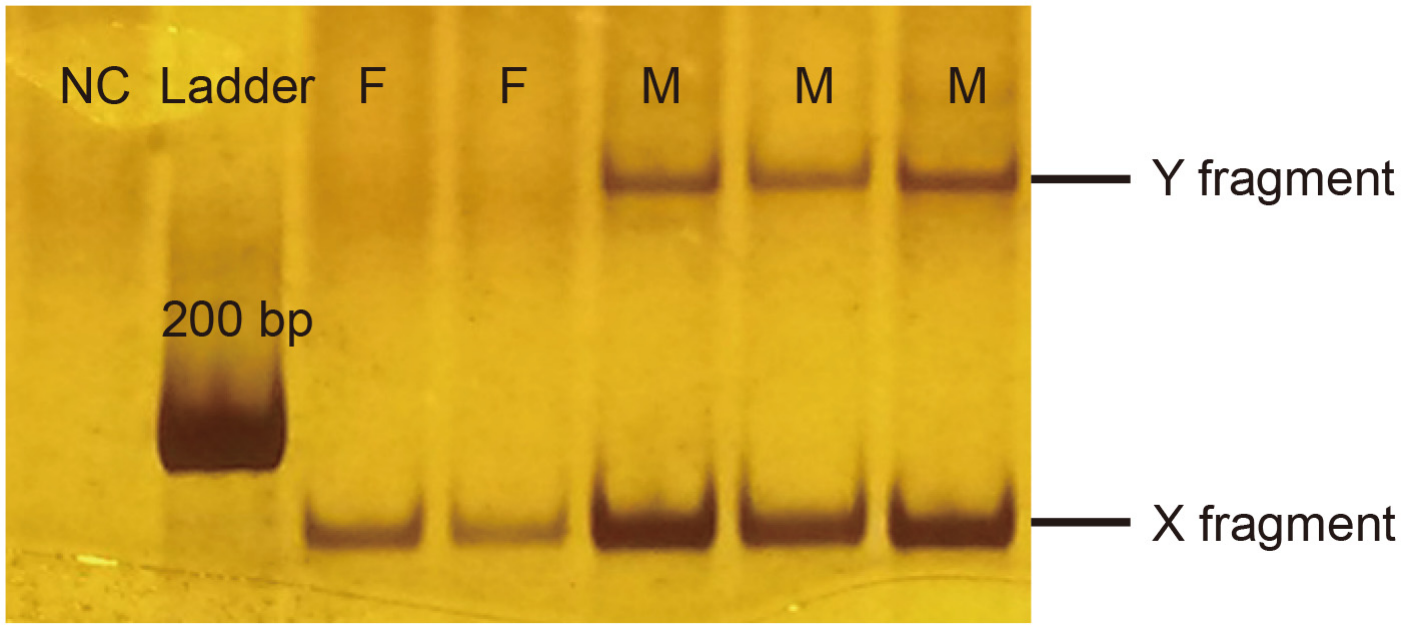
Amplification of sex chromosome-linked DEAD-box gene fragments in PCR to determine sex of fecal samples of white-headed langurs. The X- and Y-chromosome linked fragments were 178 and 208 bp in length, respectively. NC, negative control; Ladder, 200 bp DNA ladder; M, DNA isolated from feces of male white-headed langurs; F, DNA isolated from feces of female white-headed langurs.

PCR using the primers for DEAD-box gene produced a single fragment of 178 bp (the X fragment) from female DNA, and two fragments of 178 bp and 208 bp (the X and Y fragment, respectively) from male DNA in the white-headed langur (Fig 2). We were able to determine sex for 364 (91%) of all fecal samples.

### Genetic diversity of mtDNA control region

We amplified 1213 bp of the control region sequences of mtDNA from 271 fecal samples, including 178 from FS and 93 from CZ. A total of 10 nucleotide sites were variable, including nine transitions and one transversion, which defined nine different haplotypes (Hap A—Hap I, GenBank Accession No. KP772243-KP772251; Table 1). All these variable sites occurred within the first 500 bp of the amplified sequences. A fragment near the 3’ end of the control region contained five to eight AT tandem repeats in different sequences that were likely to be uninformative in population genetic analysis. Hence we only amplified the first half of the control region for the rest of the samples. A total of 390 (97%) samples from 40 social groups were successfully amplified and sequenced. To make our data comparable to other studies, we trimmed the sequences to a fragment of 350 bp (151–500 bp of the full length sequence), which encompassed the HVRI of the mtDNA control region. This fragment covered all the variable sites, thus retaining all haplotypes as the full length sequences (Table 1). Two of the haplotypes (Hap H and Hap I) that differed at one site (a C/T transition) co-occurred in all 11 samples of one group. This most likely represented a case of mitochondrial heteroplasmy.

**Table 1:** Polymorphic sites in the mtDNA HVRI sequences and number and sampling locations of the haplotypes in the white-headed langur.

**Haplotype**	**Polymorphic sites**										**FS-JCS**	**FS-BZ**	**CZ-N**	**CZ-S**	**Total population**
						1	1	1	1	3					
		3	4	4	5	1	2	2	5	2					
	9	6	6	8	1	8	1	7	2	8					
Hap A	C	G	T	T	G	A	T	T	C	T	70	0	0	0	70
Hap B				C							11	0	0	0	11
Hap C				C	A						122	0	128	24	274
Hap D	G			C	A						1	0	0	0	1
Hap E				C	A		C				4	0	0	0	4
Hap F		A		C	A						4	0	0	0	4
Hap G				C	A	G		C		C	0	15	0	0	15
Hap H			C	C	A				T		0	11	0	0	11
Hap I				C	A				T		0	11	0	0	11
****Total****											212	26	128	24	390

Vertical numbers indicate polymorphic sites in the 350 bp fragment. Blanks in sequence alignment represent identical nucleotides as Hap A. Hap H and Hap I co-occurred in 11 samples and were only counted once in total numbers. FS-JCS, Fusui-Jiuchongshan; FS-BZ, Fusui-Buzun; CZ-N, Chongzuo-North; CZ-S, Chongzuo-South.

**Fig 3:**
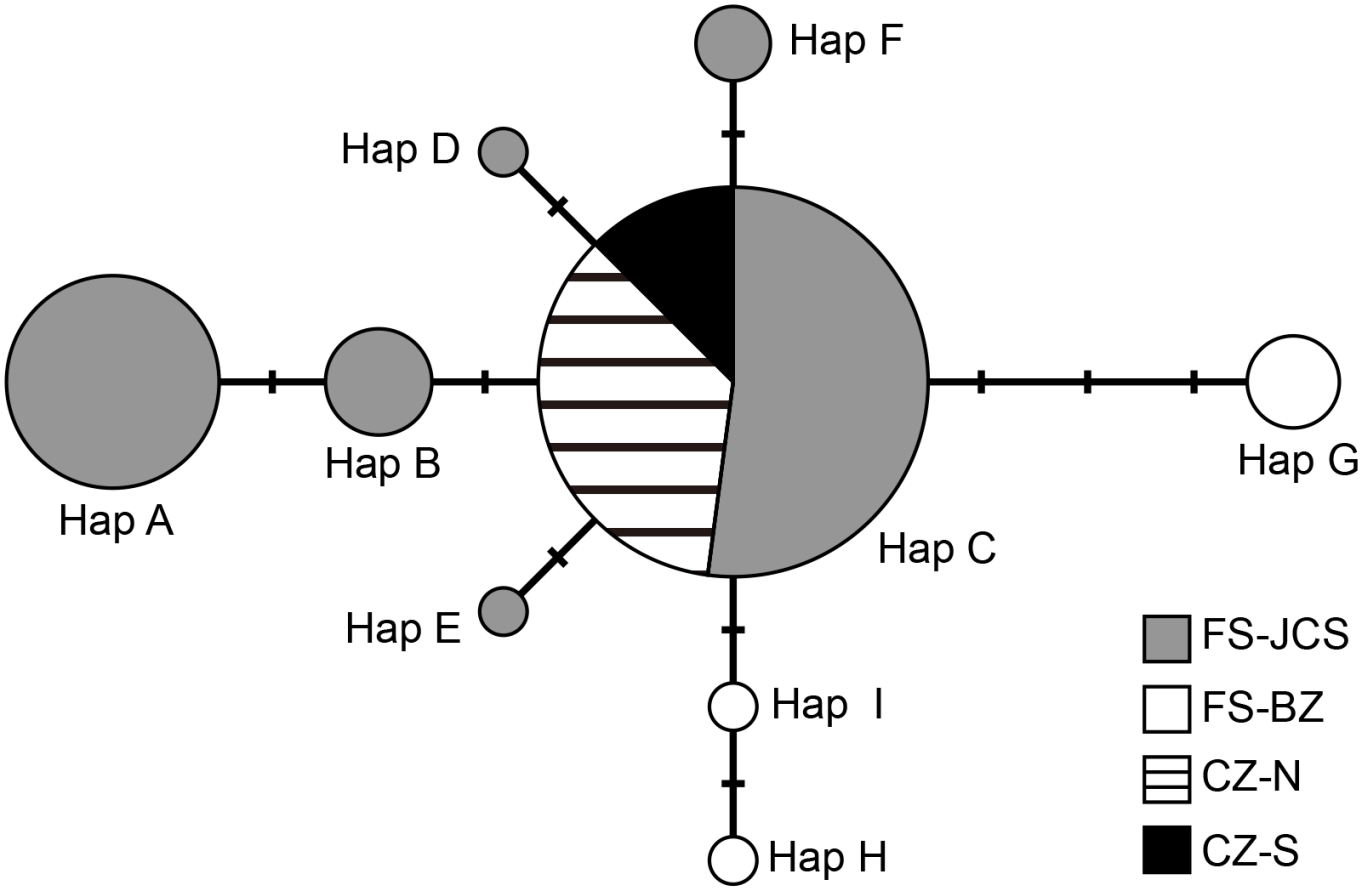
The median-joining network of mtDNA HVRI haplotypes in the FS and CZ populations of the white-headed langur. The size of each circle represents the frequency of each haplotype (N = 77), and the colors reflect the distribution area of the population. Each bar on the lines connecting two haplotypes represents one mutational step.

All nine haplotypes were present in the FS population, whereas only one haplotype (Hap C) was found in all samples from CZ. Hap C was also the most common haplotype in FS, found in 51% of all samples from FS. Additionally, haplotype distribution showed strong geographical partition within FS, with FS-JCS and FS-BZ patches sharing no haplotypes between them (Table 1; see also Fig 3).

Using a reduced sample set of 77 sequences representing different individuals from 39 social groups (see Materials and Methods), we assessed the genetic diversity parameters of the local populations (Table 2). Overall, the FS and the total populations showed very low haplotype and nucleotide diversity, and the CZ population was devoid of any genetic diversity as only a single mtDNA haplotype was found in all samples from CZ.

**Table 2:** Summary of sampling locations, sample sizes, and sequence diversity indices of the mtDNA HVRI in the white-headed langur.

**Location**	**FS-JCS**	**FS-BZ**	**All FS**	**CZ-N**	**CZ-S**	**All CZ**	**Total population**
****Sampled groups****	19	3	22	13	4	17	39
*****N*****	49	5	54	17	6	23	77
*****n*****	6	3	9	1	1	1	9
****Unique haplotypes****	5	3	8	0	0	0	-
*****s*****	5	5	10	0	0	0	10
*****h*****(*****SD*****)****	0.637 (0.048)	0.700 (0.218)	0.700 (0.046)	0	0	0	0.570 (0.056)
*****π*****(*****SD*****)****	0.00315 (0.00026)	0.00800 (0.00237)	0.00422 (0.00052)	0	0	0	0.00323 (0.00044)

*N*, number of individuals; *n*, number of haplotypes; *s*, number of polymorphic sites; *h*, haplotype diversity; *π*, nucleotide diversity. FS, Fusui; FS-JCS, Fusui-Jiuchongshan; FS-BZ, Fusui-Buzun; CZ, Chongzuo; CZ-N, Chongzuo-North; CZ-S, Chongzuo-South.

### Population genetic structure

The median-joining network of the mtDNA haplotypes indicated that all haplotypes arose from Hap C after one to three mutational events (Fig 3). The haplotypes formed a single haplogroup, and we found no substructure in the haplotype network.

The AMOVA results showed that large proportions of mtDNA variations occurred both within (62.55%) and among the local populations (37.45%) (Table 3), and uncovered a strong genetic differentiation among the populations (*Φ*_ST_ = 0.375, *P* = 0.000 ± 0.000). Additionally, all pairwise comparisons yielded significant *Φ*_ST_ values between the three populations (Table 4), indicating a strong genetic differentiation among geographical areas.

**Table 3:** Results of Analysis of Molecular Variance (AMOVA) of the mtDNA HVRI sequences in the white-headed langur.

**Source of variation**	***d*.*f*.**	**Sum of squares**	**Variance components**	**Percentage of variation**
**Among populations**	2	10.897	0.25969 Va	37.45
**Within populations**	74	32.090	0.43365 Vb	62.55
**Total**	76	42.987	0.69333	

Populations are Fusui-Jiuchongshan (FS-JCS), Fusui-Buzun (FS-BZ), and Chongzuo (CZ). *d*.*f*., degree of freedom.

**Table 4:** Pairwise comparisons of *Φ*_ST_ and *P* values between populations using the mtDNA HVRI sequences of the white-headed langur.

	**CZ**	**FS-BZ**	**FS-JCS**
****CZ****	0.00		
****FS-BZ****	0.740 (*P* = 0.00 ± 0.00)	0.00	
****FS-JCS****	0.249 (*P* = 0.00 ± 0.00)	0.535 (*P* = 0.00 ± 0.00)	0.00

FS-JCS, Fusui-Jiuchongshan; FS-BZ, Fusui-Buzun; CZ, Chongzuo.

Results of the Mantel test revealed a significant correlation between pairwise genetic distances (expressed as *Φ*_ST_/(1- *Φ*_ST_)) and geographical distances among the social groups in FS (correlation coefficient = 0.268, *P* = 0.003; S2 Table), suggesting the existence of isolation-by-distance of genetic divergence for the mtDNA sequences.

### Population demographic history

**Fig 4:**
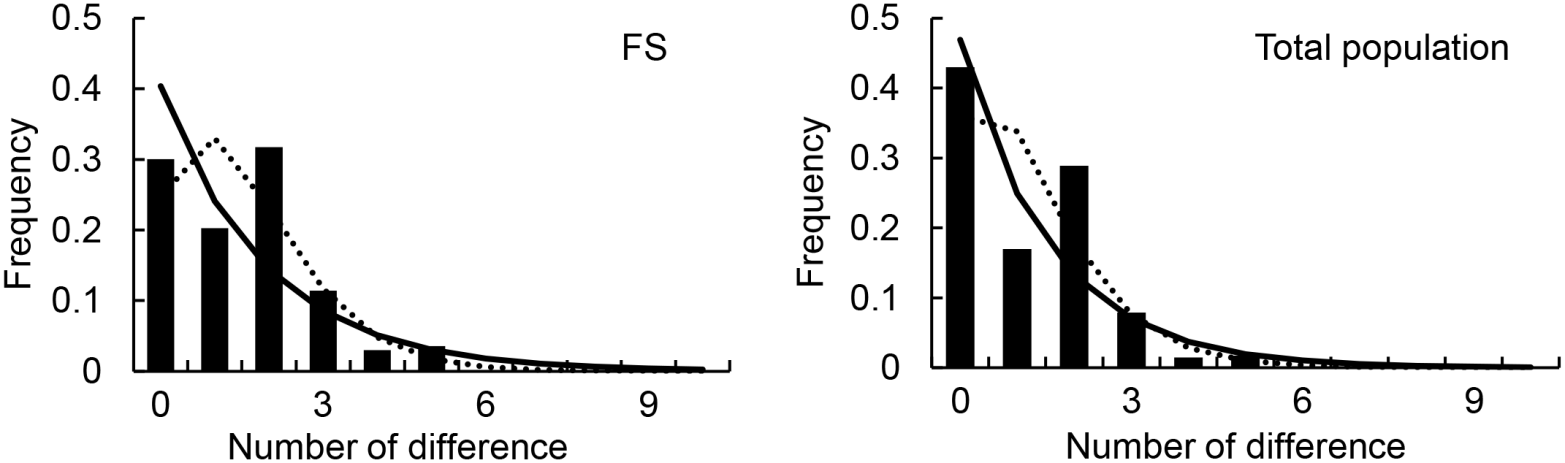
Mismatch distributions based on the mtDNA HVRI sequences of the white-headed langur. The frequencies of observed (solid bars) and expected pairwise differences under constant population size model (solid line) and population growth-decline model (dotted line) are shown. FS, Fusui.

**Fig 5:**
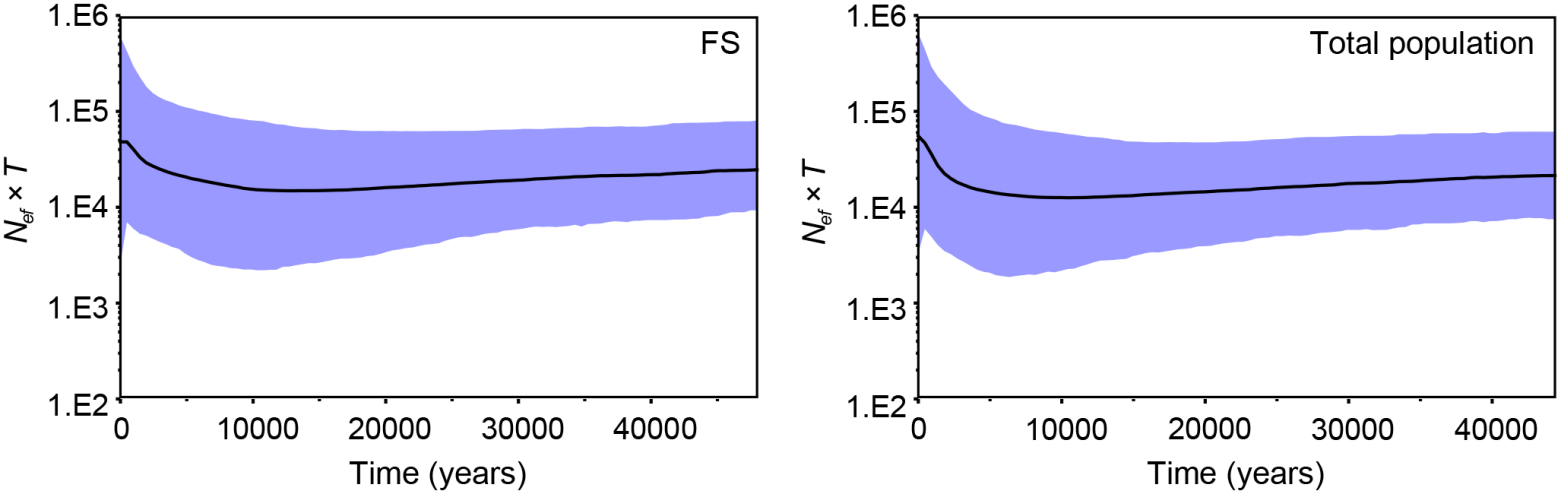
Bayesian Skyline plots showing the historical population size of the FS and total populations based on the mtDNA HVRI sequences of the white-headed langur. The X-axis is in years from present (0) to the upper estimate of the 95% highest posterior density (HPD) of time to the most recent common ancestor (TMRCA), and the Y-axis is in units of the product of female effective population size (*N*_*ef*_) and generation time (*T*). The solid line shows the median estimates, and the shaded area shows the 95% HPD limits.

We explored the demographic history of the FS and total populations. The CZ population lacked genetic diversity at the HVRI sequence and was not analyzed as a separate population. The values of Tajima’s *D* and Fu’s *F*_S_ tests for the FS and total populations were all negative but statistically nonsignificant (Table 5), suggesting demographic stability in the past. Despite the large positive values estimated for the exponential growth rate *g*, the confidence intervals (CI) were broad and included zero, indicating no or slight population expansion (Table 5). Based on the estimated population size parameter *θ*, a mutation rate of 1.64 × 10^–7^ substitutions/ site/ year for the mtDNA HVRI and a female generation time of 10 years, we calculated the *N*_*ef*_ = 3,441 (1,591–12,787 when incorporating the 95% CI for *θ*) and 3,162 (1,385–6,401; 95% CI) for the FS and total populations, respectively. The mismatch distribution analyses revealed a rugged shape of distribution for both the FS and total populations, deviating from the unimodal and smooth distribution expected under the assumption of population expansion (Fig 4). BSP analyses revealed overall constant population sizes for the FS and total populations, and only a modest population growth in the last 2,000 years (Fig 5).

**Table 5:** Results of neutrality tests and population growth rate and size estimation using the mtDNA HVRI sequences in the white-headed langur.

**Population**	**Tajima’s *D* (*P*)**	**Fu’s *F***_**S**_ **(*P*)**	***g* (95% CI)**	***θ* (95% CI)**
**FS**	-0.922 (0.191)	-2.232 (0.131)	455.00 (-182.16–3610.08)	0.011 (0.005–0.042)
**Total population**	-1.181 (0.113)	-2.808 (0.092)	568.29 (-181.52–3125.12)	0.010 (0.005–0.021)

FS, Fusui. *g*, population growth rate; *θ*, mutation-scaled effective population size. CI, confidence interval.

## Discussion

### Mitochondrial DNA diversity and population structure

The white-headed langur exhibited low genetic diversity even when compared with other highly endangered primate species with similar population sizes (e.g., *Rhinopithecus brelichi*, [55]; *Brachyteles hypoxanthus*, [56]; *Ateles geoffroyi*, [57]). In particular, the overall nucleotide diversity of the white-headed langur was an order of magnitude smaller than these other species. As the dataset (*N* = 77) used in genetic data analyses included all haplotypes identified in the total samples, increasing sampling from the total dataset can only produce even lower genetic diversity estimations. The very low genetic variation at the mtDNA level might be explained by the species’ relatively short evolutionary history, combined with a small founding population and restricted historical range. Using the mtDNA HVRI sequences, Liu et al. [21] revealed that white-headed langur haplotypes formed a monophyletic clade nested within François’ langurs, and estimated that the two lineages only split 0.46–0.27 million years ago, which is a considerably short period of time to accumulate genetic diversity for a species with a long generation time. Alternatively, the severe population decline in the last century may account for the low genetic diversity through loss of many haplotypes during population bottlenecks, and subsequent decrease in genetic variation due to random genetic drift in small populations.

As a maternally inherited genetic marker, the mtDNA is expected to show pronounced structuring in species characterized by female philopatry and male dispersal [58]. Limited female dispersal can lead to decreased mtDNA variation within breeding communities or at small spatial scales, and increased mtDNA differentiation over longer distances and among populations [59,60]. The presence of isolation-by-distance pattern of genetic divergence in mtDNA among social groups of FS is consistent with observations of strong female philopatry in this species [22,61]. However, it was unexpected that the haplotype distribution displayed such dramatic geographical specificity, with FS-JCS and FS-BZ sharing no haplotypes despite the close proximity. This phenomenon suggests that, apart from geographical distance, human activity may have a substantial impact on female dispersal which may have intensified genetic differentiation across fragmented habitat patches. Alternatively, haplotypes of the FS-BZ population may not be sufficiently represented owing to the small sample size (three groups sampled out of a total of 10 groups of the population).

Maternally inherited mtDNA only provides information for female-mediated genetic processes, whereas male-mediated gene flow is not revealed in our data. Further studies should include nuclear genetic markers to investigate genetic diversity, population structure, and gene flow patterns in this species to fully understand the species’ population genetic dynamics.

### Population history and demographic changes

In contrast to the FS population, which possessed all identified haplotypes, the CZ population only showed one haplotype in all 18 groups. This low genetic variation in mtDNA is puzzling, given the relatively large population size of CZ (over 300 individuals). Two scenarios might explain such a pattern. One is that the ancestral population first inhabited FS area, and a small number of individuals only recently dispersed to CZ and achieved the current population size in a short period of time. Since the two areas were separated by approximately 50 km of unsuitable habitat and increasing human disturbances in the recent past, further dispersal from FS to CZ was impeded. Therefore, the low genetic variation in the CZ population may be the consequence of a small founding population, restricted gene flow, and a very short population history. An alternative explanation is that the CZ and FS populations were established at a similar time and had comparable genetic diversity in the past, but the former has lost the majority of its genetic variation during severe population bottlenecks in the last century. Of an area size less than one quarter of FS, CZ is expected to have a much smaller historical population size than FS, and its genetic diversity should be more strongly influenced by population bottlenecks and genetic drift [62].

Analyses of demographic history (Tajima’s *D* and Fu’s *F*_S_ tests, population growth parameter *g*, and the BSP analyses) suggest an overall stationary historical population size and slight growth in the last 2,000 years. Since the species distribution has been confined to a narrow region with restricted suitable habitat, we expect a moderate size for the historical population, and no large-scale population expansion due to natural limits. Based on the size of the total suitable habitat (360 km^2^) and the recent census population density at CZ (13.9 individuals per km^2^; [17]), we calculate a historical population of ~5,000. Given that approximately half of the members of a social group are adult females [22], the historical female population (*N*_*f*_) is estimated to be ~2,500. Under the assumptions of an overall high and even female reproductive success and no population structure, *N*_*ef*_ is expected to approach *N*_*f*_ [63], as exemplified by a *N*_*ef*_ /*N*_*f*_ ratio close to one in certain human populations [64]. Inference of historical effective population size can be influenced by sampling schemes, population structure, marker selection, and statistical approach, and subject to large confidence intervals [48]. Nonetheless, the point estimation of the historical *N*_*ef*_ of approximately 3,000 based on genetic variation is within a reasonable range. The most likely explanation for the modest population growth in the last 2,000 years might be attributed to shrinking predator populations as a result of increasing human disturbances and habitat modification following human development in the region. The earliest historical record of the region is the establishment of the administrative division Xiangjun County in Qin Dynasty about 2,200 years ago. Natural predators of the langur such as common leopards (*Panthera pardus*) and clouded leopards (*Neofelis nebulosa*) were likely to be common in the region but have declined to near extinction probably since the time of human settlement and exploitation.

### Implications for conservation

After drastic declines in both distribution range and population size by the end of last century mainly due to habitat loss and poaching [15,19,20], populations of the white-headed langur were reported to be increasing in both FS and CZ in the last decade [17,19]. This apparent increase can be attributed to effective habitat conservation by the relevant nature reserves, reduced demands for firewood by local communities, and a strict ban on poaching since late 1990s. However, other local populations continue to shrink or become extirpated (Y. Meng, personal comm.). In addition to habitat recovery and reestablishment of local populations, genetic monitoring is essential for conservation of the species, as inbreeding effects associated with low genetic diversity can impair individual viability and adaptive potential and threaten population persistence [3,5].

According to the criteria proposed by Moritz [65], the FS-JCS, FS-BZ and CZ populations should be defined as one evolutionarily significant unit (ESU) and three management units (MUs), based on the genetic structure of the mtDNA haplotypes. The FS populations represent important genetic reservoirs for mtDNA haplotypes that are rare or absent in other local populations, whereas the CZ population displays extremely low mtDNA sequence variation. However, despite the distinct mtDNA haplotypes possessed by the FS-JCS and FS-BZ populations, we expect that there may be adequate gene flow between these patches via male dispersal owing to the close geographical proximity and lack of apparent physical barriers. The two populations are likely to be demographically connected and hence should be considered a single MU, according to the definition of MUs as demographically isolated units [66]. Based on all available evidence, we tentatively recommend recognition of two MUs corresponding to CZ and FS pending further investigation using nuclear markers.

From a planning perspective, our results emphasize the need to maintain habitat connectivity, as adjacent habitat patches can exhibit strong genetic partitions. Constructing corridors between adjoining patches, such as FS-JCS and FS-BZ, and CZ-N and CZ-S, may promote gene flow and alleviate genetic isolation due to human disturbance.

## Supporting Information

S1 Table Primer information for PCR amplifications of the white-headed langur mtDNA control region sequences. CR, control region; HVRI and HVRII, hypervariable region I and II; *T*_a_, annealing temperature.
(DOCX)

S2 Table Geographical and genetic distances between 54 social groups of the white-headed langur in Fusui (FS). Genetic distances (above diagonal) are expressed as pairwise *Φ*_ST_/(1- *Φ*_ST_) using the mtDNA HVRI sequences, and geographical distances (below diagonal) between sampling locations are in km.
(XLSX)
