# Supplementary material for: Low Genetic Diversity and Strong Geographical Structure of the Critically Endangered White-Headed Langur (Trachypithecus leucocephalus) Inferred from Mitochondrial DNA Control Region Sequences
Source: PLoS One. 2015 Jun 9;10(6):e0129782. doi: 10.1371/journal.pone.0129782 (PMC4461268; doi:10.1371/journal.pone.0129782)
Supplement: S1 Table — CR, control region; HVRI and HVRII, hypervariable region I and II; T a, annealing temperature. (DOCX) [file pone.0129782.s001.docx]

**S1 Table. Primer information for PCR amplifications of the white-headed langur mtDNA control region sequences. CR, control region; HVRI and HVRII, hypervariable region I and II; *T*_a_, annealing temperature.**

| Primer ID | CR fragment | Size (bp) | *T*_a_ (°C) | Primer sequences (5’ to 3’) |
| --- | --- | --- | --- | --- |
| P3 | HVRI | 395 | 55 | F: AACTGGCATTCTATTTAAACTAC |
| P4 |  |  |  | R: ATTGATTTCACGGAGGATGGT |
| P3 | HVRI | 574 | 55 | F: AACTGGCATTCTATTTAAACTAC |
| P470 |  |  |  | R: TGACTGGTTAATAGGGTGATAG |
| P78 | Central domain | 427 | 55 | F: CAACTTAATGTCTTCATTATCG |
| P79 |  |  |  | R: ACGTAGGTGCGGTTAATGAT |
| P82 | HVRII | 607 | 55 | F: TAACCAGTCACGGGAGCTCT |
| P87 |  |  |  | R: GGGGATGCTTGCATGTGTAA |
